# Association between serum 25-hydroxyvitamin D and prostate-specific antigen: a retrospective study in men without prostate pathology

**DOI:** 10.1515/almed-2023-0104

**Published:** 2023-10-20

**Authors:** Javier Laguna, Robin Wijngaard, Susana Hidalgo, Cristina González-Escribano, Victoria Ortiz, José Luis Bedini, Xavier Filella

**Affiliations:** Department of Biochemistry and Molecular Genetics, CDB, Hospital Clínic of Barcelona, Barcelona, Spain; CORE Laboratory, CDB, Hospital Clínic of Barcelona, Barcelona, Spain

**Keywords:** 25-hydroxyvitamin D, prostate cancer, prostate-specific antigen, vitamin D

## Abstract

**Objectives:**

Recently, vitamin D status has been associated with prostate cancer risk. However, some studies argue that there is no association of vitamin D with prostate cancer risk and serum prostate-specific antigen (PSA) concentrations. No clear conclusions can be drawn from the studies found in the literature. Our aim was to assess the relationship between PSA and 25-hydroxyvitamin D [25(OH)D].

**Methods:**

We selected 415 individuals without prostate pathologies and subgroups were generated according to age and 25(OH)D. Statistical analyses were performed using Shapiro–Wilk test, Student’s t and ANOVA tests, and Pearson’s correlation. Besides, the minimum sample size needed to obtain statistically significant results between groups according to 25(OH)D concentration was calculated and a Student’s t-test for paired samples was performed to study individuals with two PSA measurements over time, where 25(OH)D concentration increased or decreased more than 25 %.

**Results:**

We observed a slight correlation between age and PSA concentration (r=0.379, p<0.001). However, we found no significant differences when we compared PSA concentrations between groups according to 25(OH)D concentrations (p=0.891): 1.25 ± 1.32 μg/L (group with 25(OH)D<50 nmol/L) and 1.17 ± 0.90 (group with 25(OH)D≥50 nmol/L). Pearson’s correlation coefficient was close to 0. The minimum samples size to obtain statistically significant results was 815,346 men, and we observed no differences in PSA concentrations in individuals with two measurements.

**Conclusions:**

Our findings show no association in men without prostate pathologies, based on 25(OH)D levels.

## Introduction

Vitamin D is a fat-soluble vitamin obtainable from the diet (vitamin D2) or produced in the skin from 7-dehydrocholesterol under the influence of ultraviolet radiation (vitamin D3). Regardless of the source, it is metabolized to 25-hydroxyvitamin D [25(OH)D], and then to the biologically active form, calcitriol or 1,25-dihydroxyvitamin D [1,25(OH)_2_D]. The active hormone binds to the vitamin D receptor (VDR) to regulate calcium and phosphorus metabolism, which is essential for bone remodeling, reabsorption of calcium in the distal tubule and intestinal absorption of calcium and phosphorus [1, 2]. Although 1,25(OH)_2_D is the active hormone, serum concentration of 25(OH)D represents the best laboratory parameter to evaluate the vitamin D status [3].

Vitamin D deficiency has been suggested to be associated with an increased risk of multiple cancers, including prostate cancer [4]. According to the International Agency for Research on Cancer (IARC), in 2020 there were an estimated 1.41 million new cases of prostate cancer worldwide, which represents around 7 % of all cancer cases diagnosed in men. In addition, prostate cancer is the second leading cause of cancer death in men worldwide, with an estimated 375,000 deaths in 2020. It’s worth mentioning that the incidence of prostate cancer varies by age of the population. The risk of developing prostate cancer increases with age, being more common in men over the age of 50 [5].

The first study on the relationship between vitamin D deficiency and prostate cancer was published in the late twentieth century and suggested that vitamin D may protect against prostate cancer risk [6]. The EAU-EANM-ESTRO-ESUR-ISUP-SIOG guidelines on prostate cancer remarks that a U-shaped association has been observed, with both low and high vitamin D concentrations being associated with an increased risk of prostate cancer [7].

Recently, vitamin D has been proposed as a regulator of androgen intracrinology in a study using prostate cancer cells [8]. Testosterone and its 5α-reduced metabolite, dihydrotestosterone (DHT), bind to the androgen receptor (AR), a ligand-dependent nuclear transcription factor, causing tumor growth and increased expression of prostate-specific antigen (PSA), among other functions [9]. For this reason, the aim of the androgen deprivation therapy (ADT) is the decrease of testosterone and DHT concentrations. However, in some cases, the cancer no longer completely responds to the ADT and develops into a more aggressive form (castrate-resistant prostate cancer) [10]. Smith et al. [8] proposed that vitamin D inhibits the intracrine conversion of dehydroepiandrosterone (DHEA) to testosterone (and DHT). There would be no ligand for the AR, the tumor would not grow, and PSA concentration would decrease. This finding would be interesting in the treatment of the disease, especially in the castrate-resistant form.

Since then, many studies have supported the hypothesis that vitamin D concentrations are related to prostate cancer risk, even in other types of cancer [11], [12], [13], [14]. Nevertheless, other studies disagree and support that there is no association of vitamin D with prostate cancer and serum PSA concentrations [15], [16], [17], [18]. Therefore, no clear conclusions can be drawn from the studies found in the scientific literature. For this purpose, our objective was to evaluate the relationship between PSA and vitamin D, measuring 25(OH)D.

## Materials and methods

### Subjects

We conducted a retrospective study including 415 men attended in outpatient clinics of our Hospital between January 2015 and March 2021. Subjects eligible for the study were men above 18 years old (age range: 34–88 years). Exclusion criteria were patients with prostate pathology (medical history of prostate cancer, prostatitis, and benign prostatic hyperplasia), kidney failure (serum creatinine above 0.11 mmol/L) and/or liver failure (serum alanine aminotransferase, aspartate aminotransferase or gamma-glutamyl transferase above 40 U/L; or serum alkaline phosphatase above 116 U/L). During the study period, 22 % of the patients were taking vitamin D supplements as part of their daily routine.

The study was conducted in accordance with the Declaration of Helsinki (as revised in 2013) and approved by Clinical Research Ethics Committee of our hospital (Reg. HCB/2021/0631).

### Measurement of PSA and 25(OH)D

Blood samples were collected in 5 mL BD Vacutainer^®^ tubes (containing clot activator and gel for serum separation) during the early morning hours, between 8 am and 10 am. Furthermore, to account for seasonal influences, samples were collected throughout the entire year, encompassing all seasons.

Serum PSA was measured using two chemiluminescent immunoassays. From 2015 to 2019, it was measured on an ADVIA Centaur^®^ XP (Siemens Healthineers, Tarrytown, NY, USA). Since 2019, it was analyzed on an Atellica^®^ IM 1600 (Siemens Healthineers, Tarrytown, NY, USA). Serum 25(OH)D was measured using a chemiluminescent immunoassay on a LIAISON^®^ XL (DiaSorin, Saluggia, Italy).

### Statistical analysis

We generated four subgroups of individuals according to the following age intervals: 34–49 years, 50–59 years, 60–69 years and above 70 years, and two groups based on the following 25(OH)D concentrations: <50 nmol/L and ≥50 nmol/L. The threshold of 50 nmol/L (20 μg/L) was chosen according to the last recommendation [19]. In addition, we divided individuals into different groups to study the U-shaped association according to Cox proportional hazards models proposed by Kristal et al. to estimate hazard ratios for the association between plasma vitamin D and the risk of prostate cancer, with body mass index, history of diabetes, and family history of prostate cancer, included as covariates) [14].

Means and standard deviations for PSA concentrations were calculated. The Shapiro–Wilk test was used to assess the normal distribution of the variables and, if necessary, logarithmic transformations were performed. Comparison of normally distributed variables was performed using the Student’s t or ANOVA tests. A multivariate logistic regression model was performed to analyze the main considered variables (PSA levels and age) and the outcome (low or normal vitamin D levels, considered as a binary variable and using the threshold of 50 nmol/L).

We also studied the correlation between variables calculating Pearson’s correlation coefficients. Moreover, in the study of the relationship between PSA and 25(OH)D, we computed the minimum sample size needed to obtain statistically significant results between groups based on our results with a statistical power of 0.80. Besides, we performed a Student’s t-test for paired samples to study changes in PSA concentration in individuals with two measurements of PSA and 25(OH)D over time (with a minimum of six months between measurements) in which 25(OH)D increased (52 men) or decreased (22 men) more than 25 %.

All statistical analyses were performed in the R computing environment (version 4.1.3) and p-values <0.05 were considered statistically significant.

## Results

Main results regarding PSA and 25(OH)D concentration according to age are shown in Table 1. We found significant differences in PSA concentrations according to the age distributed by age groups (p<0.001), but not in 25(OH)D concentrations (p=0.409). Pairwise comparisons using t-tests showed that PSA concentrations were higher in men between 60 and 69 years and men with 70 years and older than in younger groups of men. Besides, we observed a slight correlation between age and PSA concentrations, with a r=0.379 (p<0.001).

**Table 1:** Means and standard deviations (SD) of PSA and 25(OH)D concentrations according to age.

Group	n	Age (mean ± SD), years	25(OH)D (mean ± SD), nmol/L	PSA concentration (mean ± SD), μg/L
34–49	72	44 ± 4	23.8 ± 10.3	0.67 ± 0.35
50–59	144	55 ± 3	21.7 ± 9.3	0.97 ± 0.79
60–69	137	64 ± 3	22.9 ± 10.0	1.50 ± 1.32
≥70	62	74 ± 4	24.8 ± 10.0	1.72 ± 1.38

25(OH)D, 25-hydroxyvitamin D; PSA, prostate-specific antigen.

Vitamin D deficiency (<30 nmol/L) and insufficiency (30–50 nmol/L) were observed in 185 out of 415 men (45 %), with a mean 25(OH)D concentration of 36.4 ± 8.7 nmol/L (range: 10.2–49.4 nmol/L). In the rest of the individuals, 25(OH)D concentrations were ≥50 nmol/L (range: 50.0–158.9 nmol/L). There was no statistical difference in age between groups (<50 nmol/L: 59 ± 10 years and ≥50 nmol/L: 59 ± 11 years; p=0.920). The multivariate logistic regression model did not show a significant association between 25(OH)D levels and age (p=0.239) or PSA levels (p=0.581).

We did not find significant differences when comparing PSA concentrations between the two groups based on the 25(OH)D level threshold of 50 nmol/L (1.25 ± 1.32 μg/L and 1.17 ± 0.90 μg/L, respectively, p=0.891, Table 2). Besides, we also did not find significant differences in PSA levels between men with 25(OH)D deficiency and insufficiency (1.25 ± 1.63 μg/L and 1.25 ± 1.19 μg/L, respectively, p=0.350). Regarding the study of the U-shaped relationship according to the thresholds of the models proposed by Kristal et al. [14], we have not observed a U-shaped relationship between vitamin D and PSA concentrations (Table 2).

**Table 2:** Means and standard deviations (DS) of PSA concentrations according to 25(OH)D concentration.

	Group	n	25(OH)D concentration (mean ± SD), nmol/L	PSA concentration (mean ± SD), μg/L	p-Value
According to last recommendations [19]	<50	185	36.4 ± 8.7	1.25 ± 1.32	0.891
	≥50	230	73.9 ± 20.0	1.17 ± 0.90	
Models 1 and 2 of Kristal et al. [14]	<37.5	89	28.7 ± 5.7	1.32 ± 1.63	0.955
	37.5–50	96	43.7 ± 3.5	1.19 ± 0.96	
	50–75	142	60.9 ± 6.7	1.21 ± 0.95	
	≥75	88	95.1 ± 15.7	1.09 ± 0.80	
Model 3 of Kristal et al. [14]	<44.1	141	33.1 ± 7.7	1.21 ± 1.37	0.972
	44.1–58.2	98	50.7 ± 4.0	1.23 ± 1.08	
	58.2–72.9	84	64.6 ± 4.2	1.24 ± 0.93	
	72.9–90.7	41	81.4 ± 4.5	1.13 ± 0.80	
	≥90.7	51	104.6 ± 14.5	1.11 ± 0.80	

25(OH)D, 25-hydroxyvitamin D; PSA, prostate-specific antigen.

**Figure 1:**
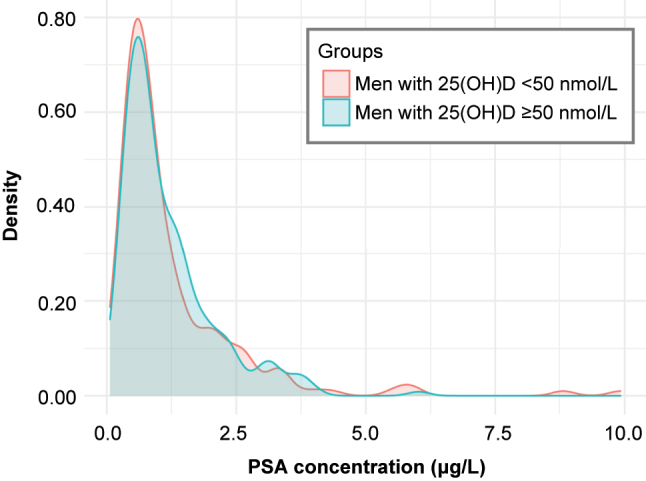
Distribution of PSA concentrations in men according to 25(OH)D concentrations. 25(OH)D, 25-hydroxyvitamin D; PSA, prostate-specific antigen.

Figure 1 shows the distribution of serum PSA concentrations according to the 25(OH)D group. We observed that both groups almost overlap in their distribution due to the data similarity. Pearson’s correlation coefficient was close to 0 (r=−0.001; p=0.985), indicating that there was no linear relationship between PSA and 25(OH)D concentrations.

The minimum sample size needed to obtain statistically significant results between men presenting 25(OH)D<50 nmol/L and men with 25(OH)D≥50 nmol/L was computed. A Cohen’s d of 0.004 was obtained, indicating a very small effect size between 25(OH)D groups. The sample size calculation with a power of 0.80 showed that we needed 815,346 men in each group in order to find statistically differences.

Furthermore, changes in PSA concentrations were studied in individuals with two measurements of PSA and 25(OH)D over time. We did not find statistically significant differences in PSA concentrations between measurements in which 25(OH)D increased more than 25 % (1.10 ± 0.97 μg/L and 1.13 ± 0.84 μg/L, p=0.178) or decreased more than 25 % (0.96 ± 1.16 μg/L and 0.98 ± 1.25 μg/L, p=0.533).

## Discussion

It is well-known that PSA is the most widely used tumor marker for the detection and monitoring of patients with prostate cancer [20]. However, PSA is not cancer-specific, so it may be elevated in other non-malignant conditions [7]. Some studies have reported the importance of a good vitamin D status to prevent development and progression of prostate cancer [3], so we decided to carry out the present work to study this relationship in men without prostatic pathologies or liver or kidney failure. The hypothesis of this work is that vitamin D deficiency may lead to an increase in PSA for two reasons: 1) because low vitamin D concentrations induce prostate cancer and, therefore, in the population with low vitamin D concentrations there are more individuals with undetected prostate cancer than when vitamin D is high and 2) because low vitamin D concentrations increase PSA.

We observed a slight correlation between PSA and age. The lowest PSA concentrations belonged to the younger patient groups (34–49 years and 50–59 years) and the highest concentrations were observed in individuals with 60–69 years and ≥70 years, in accordance with the data published by Oesterling et al. [21]. In contrast, no association was found between age and 25(OH)D levels. Although it has been believed that vitamin D levels decrease as people age, studies have found that there is no significant difference in vitamin D levels among people of different ages. An explanation could be the inclusion of patients under 75 years old in these studies [22]. Our study included males between the ages of 34 and 88 years, but only 10 patients were above the age of 75. This might explain the lack of association between 25(OH)D levels and age.

Moreover, we observed no relationship between PSA and 25(OH)D concentrations in our study population. No statistically significant differences were observed when we compared men with vitamin D deficiency and men with 25(OH)D concentrations ≥50 nmol/L. Similarly, Tóth et al. [15] retrospectively studied a cohort of 5,136 men, observing no difference in PSA concentrations between individuals with different vitamin D concentrations. Besides, in assessing cancer risk, no association has been found between vitamin D levels and prostate cancer risk [17, 18]. However, Kristal et al. observed a U-shaped association between 25(OH)D concentrations and prostate cancer risk. They observed that individuals with low or high 25(OH)D concentrations had an increased risk of cancer, especially high-grade disease [14]. However, we did not observe differences in PSA concentration between the groups and, therefore, men with low and high 25(OH)D concentrations did not have higher PSA concentrations than the rest of groups.

It should be noted that we included men with normal creatinine and hepatic enzymes concentrations. This is an aspect that is not considered in other studies. Serum PSA concentrations are influenced by liver disease, and it has been demonstrated that cirrhotic patients have lower serum PSA concentrations than noncirrhotic men [23]. Besides that, vitamin D deficiency is common among patients with chronic kidney or liver disease [24, 25]. For these reasons, the inclusion of these individuals could lead to misleading conclusions.

We hypothesized that vitamin D deficiency would lead to elevated PSA concentrations, either as a consequence of the direct effect of vitamin D on PSA synthesis or because of the presence of men with undiagnosed prostate cancer. We based our study on one 25(OH)D measurement, which reflects vitamin D stores at a single point. In fact, this is a weakness in most of the studies conducted on this issue. Cancer is a long-term process, so seeing an association would be very difficult with a single vitamin D measurement.

In contrast, studying the direct effect of vitamin D is easier to assess. In this study, we have observed that there are no differences in PSA concentrations between individuals with different 25(OH)D concentrations. Furthermore, we studied men with two measurements of PSA and 25(OH)D over time, with a significant change in the 25(OH)D concentration. These measurements were analyzed by paired samples t-tests and we did not observe statistically significant differences between them. In 2014, Chandler et al. [26] performed a prospective study in 105 men to evaluate the impact of vitamin D supplementation on PSA concentrations, randomly distributing individuals into four groups (placebo, 1,000, 2,000 and 4,000 IU of vitamin D) over a three-month period. In the group of men treated with 4,000 IU of vitamin D, mean 25(OH)D concentrations at baseline and at three months were 44.4 and 118.0 nmol/L, respectively. However, this increase in the 25(OH)D concentration did not influence PSA concentrations, which remained stable (2.15 and 2.20 μg/L at baseline and at three months, respectively). Therefore, given the current understanding, there is no compelling evidence to suggest that the use of vitamin D supplements influences PSA levels.

Regarding the study limitations, we cannot rule out that there is an influence of 1,25(OH)_2_D on PSA concentration, so the 1,25(OH)_2_D measurement should be considered in further studies. 1,25(OH)_2_D is the active agonist of VDR and it regulates cellular differentiation and proliferation of many cell types, such as prostate epithelial cells [27, 28]. Consequently, 1,25(OH)_2_D concentrations have been thought to be related to prostate cancer risk and PSA concentration [29, 30].

In conclusion, although other studies have suggested that vitamin D influences serum PSA concentrations, our findings show no association in men without prostatic pathologies and liver or kidney failure, based on 25(OH)D levels. Furthermore, additional studies are needed to corroborate the role of 1,25(OH)_2_D on PSA concentration.
